# Supplementary material for: Redox imbalance dictates dependence on GOT1 versus GOT2 for rod photoreceptor health during aging and stress
Source: bioRxiv. 2026 Apr 7:2026.04.05.716322. Preprint. [Version 1] doi: 10.64898/2026.04.05.716322 (PMC13081828; doi:10.64898/2026.04.05.716322)
Supplement: 1 [file NIHPP2026.04.05.716322V1-supplement-1.pdf]

bioRxiv preprint doi: https://doi.org/10.64898/2026.04.05.716322; this version posted April 7, 2026. The copyright holder for this preprint (which was not certified by peer review) is the author/funder, who has granted bioRxiv a license to display the preprint in perpetuity. It is made available under aCC-BY 4.0 International license.

Supplementary

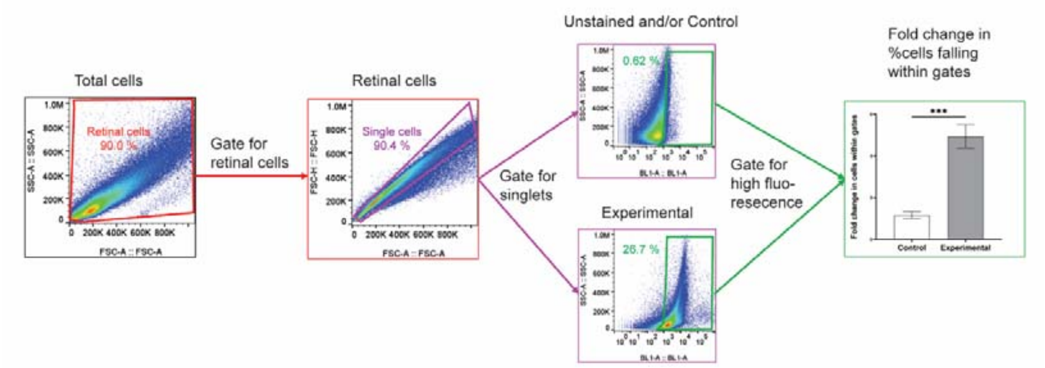

Supp. Fig. 1: Flow cytometry gating strategy. Cells were first gated by forward and side scatter to exclude debris and broken cells (red gate), followed by selecting singlets using an FSC-A *vs.* FSC-H plot (purple gate). Singlets within the purple gate were then analyzed for a change in fluorescent intensity for each sample (green gate). The fold change in the percentage of cells within the green gate was used to

bioRxiv preprint doi: https://doi.org/10.64898/2026.04.05.716322; this version posted April 7, 2026. The copyright holder for this preprint (which was not certified by peer review) is the author/funder, who has granted bioRxiv a license to display the preprint in perpetuity. It is made available under aCC-BY 4.0 International license.

determine changes in TUNEL staining.

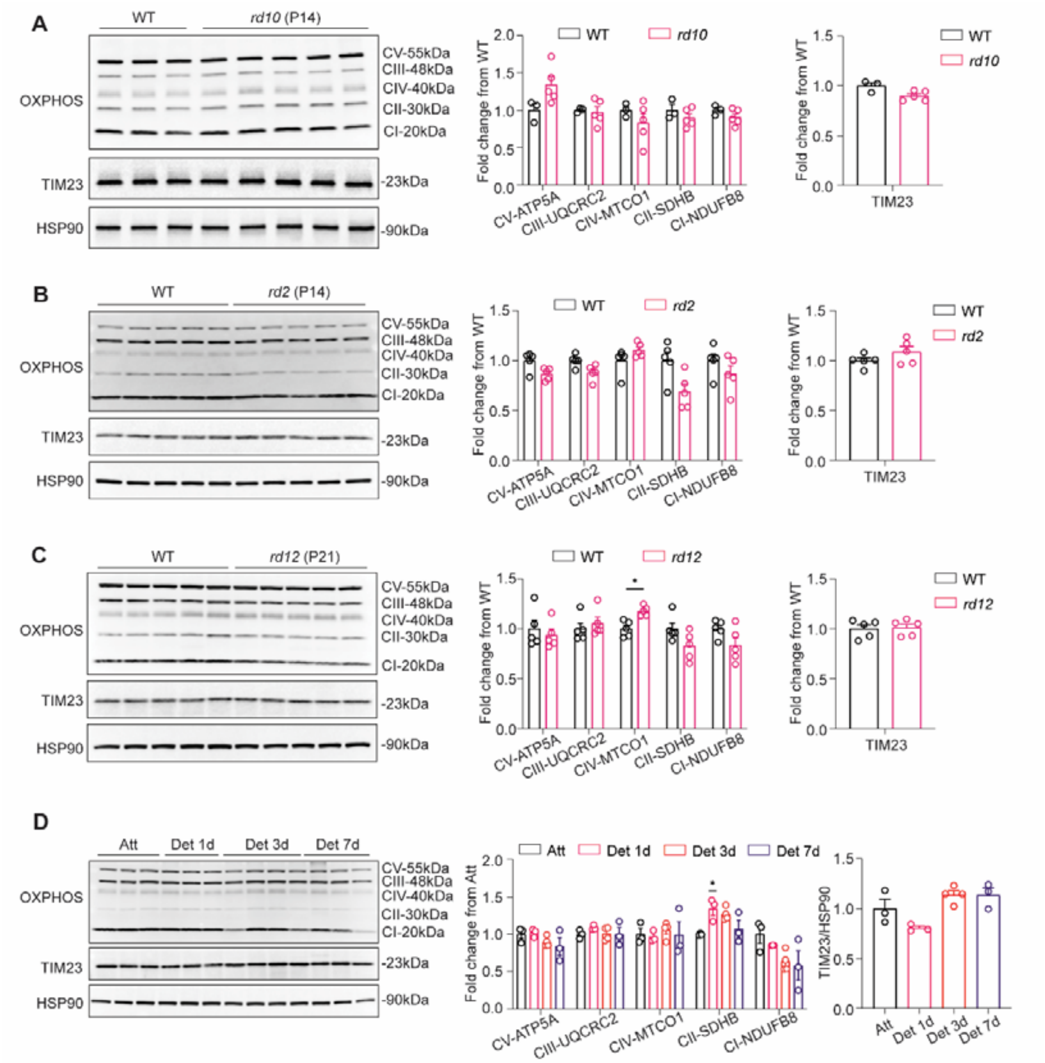

Supp. Fig. 2. Retinal mitochondrial oxidative phosphorylation complexes and TIM23 remain unchanged in different PR stress models. (A-C) Western blot analysis and quantitation of oxidative phosphorylation (OXPHOS) complexes (CV-ATP5A, CIII-UQCRC2, CIV-MTCO1, CII-SDHB, CI-NDUFB8) and TIM23 show no differences between *rd10* (P14), *rd2* (P14), *rd12* (P21) and age-matched wild-type (WT) retina. **(D)** No significant downregulation was observed in the expression of OXPHOS complexes and mitochondrial protein TIM23 in the retinas 1, 3, and 7 days after retinal detachment (Det) compared to attached (Att) retinas. N=3-5 animals per group; Statistical differences in (A-C) are based on an unpaired two-tail student's T-test, and (D) are based on one-way ANOVA as compared to WT retina; *P <0.05. Mean ± SEM. CI-NDUFB8: complex 1, NADH:ubiquinone oxidoreductase subunit B8; CII-SDHB: complex 2, succinate dehydrogenase complex iron sulfur subunit B; CIII-UQCRC2: complex 3, ubiquinol-cytochrome c reductase core protein 2;

bioRxiv preprint doi: https://doi.org/10.64898/2026.04.05.716322; this version posted April 7, 2026. The copyright holder for this preprint (which was not certified by peer review) is the author/funder, who has granted bioRxiv a license to display the preprint in perpetuity. It is made available under aCC-BY 4.0 International license.

CV-ATP5A: complex 5, ATP synthase F1 subunit alpha; TIM23: translocase of the inner membrane 23; HSP90: heat shock protein 90.

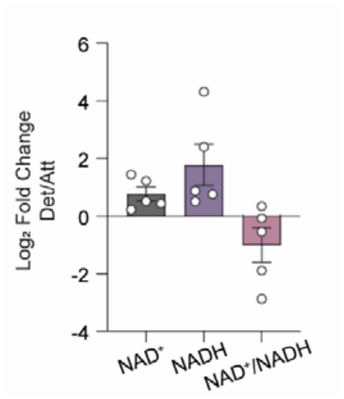

Supp. Fig. 3. Trend toward NADH accumulation in detached compared to attached retina. Log_2 fold change in detached versus attached retinal levels of NAD+ and NADH as well as the NAD^+/NADH ratio after 24 hours. Two attached or detached retinas were pooled for each N with N=5 for both the attached and detached mouse groups. NAD^+: nicotinamide adenine dinucleotide, NADH: nicotinamide adenine dinucleotide + hydrogen.

Supp. Table 1. Gene expressions changes in *Got1* cKO and *Got2* cKO versus WT retina at 2 months of age.

Pathway	Gene Abbreviation	Forward Primer	Reverse Primer	*Got1* cKO[1][a]		*Got2* cKO	
				Fold Change	P-value	Fold Change	P-value
**Reference Gene**	*Actb*	AGCCATGTACGTAGCCATCC	CTCTCAGCTGTGGTGGTGAA	N/A	N/A	N/A	N/A
**Malate-Aspartate Shuttle**	*Got1*	AGAGAAAGATGCGTGGGCTA	TGGACCAGGTGATTCGTACA	0.88	0.461	1.25	0.004
	Got2	GTTGAAATGGGACCTCCAGA	GGGCAGGTATTCTTTGTCCA	1.05	0.620	0.40	<0.000001
	Mdh1	GAAGCCCTGAAAGACGACAG	TCGACACGAACTCTCCCTCT	0.57	0.011	2.10	0.010
	Mdh2	GCTTTGTCTTCTCCCTCGTG	CAAAGTCCTCGCCTTTCTTG	0.55	0.014	1.50	0.069
**Glycolysis**	*Hk2*	GGGACGACGGTACACTCAAT	GCCAGTGGTAAGGAGCTCTG	0.90	0.736	1.03	0.840
	Pfkl	AACATTTACAAGCTCCTCGCCC	CAGCCTACTTCTTGCACCTGAC	1.47	0.016	0.76	0.020
	Pkm2	ATTACCAGCGACCCCACAGAA	ACGGCATCCTTACACAGCACA	0.92	0.508	1.19	0.084
	Pkm1	ATAGCTCGGGAGGCTGAGGCA	GACTCCGTGAGAACTATCAAA	0.99	0.894	1.00	0.977
	Ldha	GTTGTTGGGGTTGGTGCTGT	TCATCTCGCCCTTGAGTTTG	1.02	0.906	0.78	0.133
	Glut1	TCAACACGGCCTTCACTG	CACGATGCTCAGATAGGACATC	0.91	0.633	0.83	0.131
**Pyruvate Metabolism**	*Pdk1*	GGCGGCTTTGTGATTTGTAT	ACCTGAATCGGGGGATAAAC	0.75	0.096	0.83	0.084
	Pdk2	AGGAAGTCAATGCCACCAAC	GAGGGCCACCATAATCTTGA	0.95	0.785	1.03	0.581
	Pdk3	GTGGAGTCCCACTTCGAAAA	AAAACTGGCAGCCTCTCAA	0.68	0.041	1.76	0.021
	Pdha1	GGGACGTCTGTTGAGAGAGC	TGTGTCCATGGTAGCGGTAA	0.87	0.177	1.03	0.535
	Pdhb	TCGAAGCCATAGAAGCCAGT	AGGCATAGGGACATCAGCAC	0.59	0.021	1.48	0.002
	Pcx	ATGTTGTGGACGTGGCAGTA	AATCGAAGGCTGCGTACAGT	1.05	0.807	0.91	0.207
	Me1	GGGATTGCTCACTTGGTTGT	GTTCATGGGCAAACACCTCT	0.86	0.069	1.17	0.487
	Me2	TTGTGTTCCCTGCATGGTTA	ATACAGACGGGCAGACCAAC	1.05	0.798	1.51	0.019
**Tricarboxylic Acid (TCA) Cycle**	*Cs*	TGCCTAAGGATCCCATGTTC	TTCATCTCCGTCATGCCATA	1.21	0.245	1.27	0.079
	Aco1	GGGTGATCCCCCTTGAGTAT	GATGCCTCCATTGTGGAAGT	1.18	0.512	1.45	0.001
	Aco2	CAACATGGGTGCAGAAATTG	GTGAGCCAAGTCAGGGGTAA	0.91	0.242	0.96	0.344
	Idh3a	GAGGTTTTGCTGGTGGTGTT	TCCTCCTGGTCCTTGAATTG	2.78	0.029	1.08	0.347

bioRxiv preprint doi: https://doi.org/10.64898/2026.04.05.716322; this version posted April 7, 2026. The copyright holder for this preprint (which was not certified by peer review) is the author/funder, who has granted bioRxiv a license to display the preprint in perpetuity. It is made available under aCC-BY 4.0 International license.

	Idh3b	ATCTGAGCGAGGTGCAGAAT	TACGTTGGCAAACAAATCCA	0.85	0.084	1.14	0.200
	Idh3g	TGTAAGCTCCAACGCTGATG	CACTCCTGGCAGGCTCTTAC	0.78	0.038	1.37	0.003
	Sucla2	TTGTGCATGGATGCAAAGAT	CCAAGCCAGCACCATTTACT	0.72	0.083	1.57	0.001
	Suclg1	AGATTCCCTTGGTTGTGTGC	GGGTTGTTTGGTGAACTGCT	0.73	0.006	1.37	0.040
	Suclg2	CTTTGGTGGGATCGTCAACT	AACAGCTTTCTTGGCTGCAT	0.77	0.070	1.41	0.203
	Sdha	ACACAGACCTGGTGGAGACC	GGATGGGCTTGGAGTAATCA	0.69	0.026	1.41	0.008
	Sdhb	ACTGGTGGAACGGAGACAAG	TTAAGCCAATGCTCGCTTCT	0.64	0.025	1.74	0.007
	Sdhc	GGAGGGGTCTCTCTTTTTGG	AAGTGTCGGATCCCATTCAG	0.80	0.022	1.47	0.017
	Sdhd	GATCCCTGCTGGGTACTTGA	AAGTAGCAAAGCCCAGCAAA	0.76	0.003	1.46	0.032
	Fh1	AGCAATGCATATTGCTGCTG	CGCATACTGGACTTGCTGAA	0.37	0.004	1.60	0.006
**Amino Acid Metabolism**	*Bcat1*	TAGAATGTGCCGATCTGCTG	CTTTGGAAGGCTTCTTGACG	0.98	0.784	1.35	0.075
	Bcat2	GGACCCATGAAGACGGAGTA	CCCGCTTCAATTCCTTCATA	0.74	0.018	1.59	0.006
	Gls	CGTTCCATGTTGGTCTTCCT	ATCACCGACTTCACCCTTTG	1.00	0.998	1.36	0.085
	Gls2	GACCGTGGTGAACCTGCTAT	ACCTCCAGGTGGTTGAACTG	0.60	0.004	1.62	0.021
	Asns	ATTACGACAGTTCGGGCATC	TCTCAGTTCGAGACCGTGTG	0.57	0.0003	1.89	0.004
**Redox Balance**	*Gsr*	CACGACCATGATTCCAGATG	CAGCATAGACGCCTTTGACA	0.79	0.097	1.31	0.108
	Gss	GCCTCCTACATCCTCATGGA	CCACATGCTTGTTCATCACC	0.81	0.445	2.44	0.055
	Gpx1	ATCAGTTCGGACACCAGGAG	CATTCCGCAGGAAGGTAAAG	0.63	0.005	1.56	0.005
	Gpx4	CCGGCTACAACGTCAAGTTT	ACGCAGCCGTTCTTATCAAT	0.66	0.001	1.50	0.011
	G6pdx	CCTACCATCTGGTGGCTGTT	CATTCATGTGGCTGTTGAGG	1.08	0.337	0.83	0.050
	Pgd	GGGCACTTTGTGAAGATGGT	AACAGCTCTTTGCCGTCAGT	3.69	0.001	1.54	0.022
	Mthfd1	AGCACAGTAGAGAGCGCACA	CAGGCGATCTAATGCTGACA	1.04	0.579	1.09	0.602
	Idh1	AGGTTCTGTGGTGGAGATGC	GACGCCCACGTTGTATTTCT	0.76	0.048	1.07	0.237
	Idh2	CCGTCTTCAGAGAGCCAATC	GAAATGGACTCGTCGGTGTT	1.14	0.237	1.27	0.246
	Sod1	CCAGTGCAGGACCTCATTTT	CACCTTTGCCCAAGTCATCT	0.54	0.025	1.84	0.002
	Sod2	CCGAGGAGAAGTACCACGAG	GCTTGATAGCCTCCAGCAAC	0.88	0.058	1.53	0.008
	Cat	ACATGGTCTGGGACTTCTGG	CAAGTTTTTGATGCCCTGGT	1.12	0.318	1.49	0.007
**Cell Death**	*Casp3*	GGGCCTGTTGAACTGAAAAA	CCGTCCTTTGAATTTCTCCA	1.00	0.984	1.01	0.909

bioRxiv preprint doi: https://doi.org/10.64898/2026.04.05.716322; this version posted April 7, 2026. The copyright holder for this preprint (which was not certified by peer review) is the author/funder, who has granted bioRxiv a license to display the preprint in perpetuity. It is made available under aCC-BY 4.0 International license.

**Pathways**	*Casp8*	CCTAGACTGCAACCGAGAGG	GCAGGCTCAAGTCATCTTCC	4.36	0.043	0.94	0.619
	Casp9	GATGCTGTCCCCTATCAGGA	GGGACTGCAGGTCTTCAGAG	2.05	0.155	0.88	0.485
	Ripk1	CCTGCTGGAGAAGACAGACC	CATCATCTTCCCCTCTTCCA	1.48	0.433	0.77	0.069
	Atg5	AGATGGACAGCTGCACACAC	GCTGGGGGACAATGCTAATA	0.45	0.085	1.19	0.314
	Sqstm1	GCTCAGGAGGAGACGATGAC	AGAAACCCATGGACAGCATC	0.96	0.621	1.11	0.322
	Gch1	CACCAAGGGATACCAGGAGA	AGCCAATATGGACCCTTCCT	0.58	0.014	0.84	0.188
	Fth1	CGAGATGATGTGGCTCTGAA	GTGCACACTCCATTGCATTC	0.62	0.021	1.18	0.180
	Hmgcr	TGGAGATCATGTGCTGCTTC	GCGACTATGAGCGTGAACAA	1.25	0.173	0.92	0.566
	Chac1	GTACGGCTCCCTAGTGTGGA	GTCTTCAAGGAGGGTCACCA	0.97	0.833	0.98	0.913
	Ptgs2	AGAAGGAAATGGCTGCAGAA	GCTCGGCTTCCAGTATTGAG	1.08	0.873	1.04	0.825
	Rpl8	AAGCGGACAGAGCTGTTCAT	CTGGGTTGTGGGAGATGACT	0.83	0.299	1.11	0.371

[1] S. Subramanya, M.T. Goswami, N. Miller, E. Weh, S. Chaudhury, L. Zhang, A. Andren, H. Hager, K.M. Weh, C.A. Lyssiotis, C.G. Besirli, T.J. Wubben, Rod photoreceptor-specific deletion of cytosolic aspartate aminotransferase, GOT1, causes retinal degeneration, Front. Ophthalmol. 3 (2023). https://doi.org/10.3389/fopht.2023.1306019.

[a] Relative mRNA expression data for *Got1* cKO mice was reused with permission. Copyright © 2023 Subramanya, Goswami, Miller, Weh, Chaudhury, Zhang, Andren, Hager, Weh, Lyssiotis, Besirli and Wubben.

bioRxiv preprint doi: https://doi.org/10.64898/2026.04.05.716322; this version posted April 7, 2026. The copyright holder for this preprint (which was not certified by peer review) is the author/funder, who has granted bioRxiv a license to display the preprint in perpetuity. It is made available under aCC-BY 4.0 International license.
